# Identifying hotspots of faunal data deficiency to direct urgent research and monitoring

**DOI:** 10.1111/cobi.70341

**Published:** 2026-06-04

**Authors:** Matt W. Hayward, Danielle Verdon‐Kidd, K. Colyvas, Ellen Saxon, Andrea S. Griffin

**Affiliations:** ^1^ School of Science University of Newcastle Callaghan New South Wales Australia; ^2^ Mammal Research Institute University of Pretoria Tshwane South Africa; ^3^ Centre for African Conservation Ecology Nelson Mandela University Port Elizabeth South Africa

**Keywords:** hotspots, IUCN Red List, status assessment, threatened species, especie amenazada, evaluación de estado, Lista Roja de la UICN, puntos calientes

## Abstract

Data deficiency is a substantial challenge for extinction risk assessments because incomplete data means we cannot accurately identify priority protected areas for conservation. Here, we use the International Union for Conservation of Nature (IUCN) distributions of amphibians, sharks and rays, fish, mammals, birds, and reptiles to identify hotspots of data deficient species. We found that areas with high numbers of data deficient species were not randomly distributed and did not necessarily overlap with hotspots of biodiversity, particularly in the Amazon and West Africa. Data deficiency hotspots were concentrated around tropical South America, Africa, and Southeast Asia; however, this distribution varied between taxa. Hotspots of data deficient amphibians were concentrated around the tropical Andean forests of Colombia, Peru, and Ecuador; throughout Brazil; and in the New Guinea Highlands. The tropical forests of the Congo River basin, Papua New Guinea, and the Philippines were hotspots for avian data deficiency. Data deficient chondrichthyans were concentrated in the tropical seas, particularly around the western coast of Africa, East Asia, and the tropical Americas. Indochina was identified as the primary hotspot for data deficient freshwater fish, whereas the Indo‐Pacific was the primary hotspot for data deficient marine fish. Data deficient marine mammals were broadly distributed around the southern oceans, South America, Southeast Asia, and Northwest Africa. Data deficient herpetofauna were concentrated around tropical Asia, whereas tropical South America, Indonesia, and central Africa were the hotspots for data deficient terrestrial mammals. Although data deficiency reflected drivers of biodiversity more broadly (e.g., latitude), other factors were also influential, including the amount of conservation funding invested by a country and population density. Increasing evidence suggests that data deficient species are disproportionately likely to be threatened with extinction. Scientists urgently need to focus their research efforts on these data deficiency hotspots and ensure protected area networks are suitably representative and include data deficient species. Furthermore, the accuracy of status assessments needs to be ascertained, and data deficient species need to be prioritized for study to ensure they are not lost before they are identified.

## Abstract (es)

Identificación de puntos calientes de deficiencia de datos faunísticos para dirigir la investigación y el monitoreo urgentes

**Resumen:**

La falta de datos es un reto importante para las evaluaciones del riesgo de extinción, ya que la información incompleta nos impide identificar con precisión las áreas protegidas prioritarias para la conservación. En este estudio, utilizamos los datos de distribución de la Unión Internacional para la Conservación de la Naturaleza (UICN) sobre anfibios, tiburones y rayas, peces, mamíferos, aves y reptiles para identificar los puntos críticos de especies con datos insuficientes. Descubrimos que las áreas con un elevado número de especies con datos insuficientes no se distribuían de forma aleatoria y no se solapaban necesariamente con los puntos calientes de biodiversidad, especialmente en la Amazonía y África Occidental. Los puntos calientes de especies con datos insuficientes se concentraban en torno a las zonas tropicales de Sudamérica, África y el sudeste asiático; sin embargo, esta distribución variaba entre los taxones. Los puntos calientes de anfibios con datos insuficientes se concentraban en torno a los bosques tropicales andinos de Colombia, Perú y Ecuador; en todo Brasil; y en las tierras altas de Nueva Guinea. Los bosques tropicales de la cuenca del río Congo, Papúa Nueva Guinea y Filipinas eran puntos calientes para las aves con datos insuficientes. Los condrictios con datos insuficientes se concentraban en los mares tropicales, especialmente en torno a la costa occidental de África, el este de Asia y las zonas tropicales de América. Se identificó Indochina como el principal punto caliente para los peces de agua dulce con datos insuficientes, mientras que el Indo‐Pacífico era el principal punto caliente para los peces marinos con datos insuficientes. Los mamíferos marinos con datos insuficientes se distribuían ampliamente por los océanos del sur, Sudamérica, el sudeste asiático y el noroeste de África. La herpetofauna con datos insuficientes se concentraba en la zona tropical de Asia, mientras que la zona tropical de Sudamérica, Indonesia y África central eran los puntos calientes para los mamíferos terrestres con datos insuficientes. Si bien la falta de datos reflejaba factores que influyen en la biodiversidad de manera más general (por ejemplo, la latitud), también influían otros factores, como la cantidad de fondos destinados a la conservación por parte de un país y la densidad de población. Cada vez hay más indicios de que las especies con datos insuficientes tienen una probabilidad desproporcionadamente mayor de estar en peligro de extinción. Los científicos deben centrar urgentemente sus esfuerzos de investigación en estos puntos calientes de falta de datos y garantizar que las redes de áreas protegidas sean adecuadamente representativas e incluyan a las especies con datos insuficientes. Además, es necesario verificar la precisión de las evaluaciones de estado, y se debe dar prioridad al estudio de las especies con datos insuficientes para garantizar que no se pierdan antes de ser identificadas.

## INTRODUCTION

A lack of species occurrence data poses a significant challenge to assessing extinction risk. When crucial information regarding a species’ abundance, distribution, or population trends is unavailable, conservation assessors cannot easily assign a threat status category on platforms such as the International Union for Conservation of Nature (IUCN) Red List (Mace & Lande, 1991) or national lists of endangered species. Consequently, conservation managers cannot confidently and accurately determine whether management interventions are needed or what management would be most effective. Concerningly, 15% (16,355) of the 112,432 IUCN‐assessed species are listed as data deficient on the IUCN Red List (IUCN, 2018); however, only 8% of the world's described species have had their status assessed (https://www.iucnredlist.org/about/barometer‐of‐life). Hence, there is a large degree of uncertainty over the level and location of data deficient species, which “may be well studied, and [their] biology well‐known, but appropriate data on abundance or distribution are lacking” (IUCN, 2012). Information on distribution and abundance is fundamental to extinction risk assessments as well, as four of the five criteria used to assess the status of species involve these two factors (IUCN, 2012).

There are likely many more data deficient species than have been identified, and therefore, there are likely many more threatened species. It has been suggested that data deficient reptiles will be threatened in a similar proportion to better known reptiles (Bland & Böhm, 2016); however, there are likely to be vastly more data deficient terrestrial mammals that are ultimately assessed as threatened (Bland et al., 2015). More concerningly, machine learning–derived status assessments for 7699 data deficient species suggest they may be much more threatened than data sufficient taxa, with 88% of data deficient amphibians and more than half of data deficient mammals and reptiles likely to be threatened (Borgelt et al., 2022). Furthermore, more than half of all data deficient amphibians have less than 1% of their estimated distribution within protected areas, whereas 58% of their distributions overlap with human‐modified landscapes (Nori & Loyola, 2015), reflecting the substantial threats these species are likely to face.

One method that conservationists have used to identify important locations for establishing protected areas and implementing other conservation actions is to identify biodiversity hotspots (Hanson et al., 2020; Jenkins et al., 2013; Tilker et al., 2020). Hotspots are areas featuring exceptional biodiversity, endemic species concentrations, or habitat loss (Ginsberg, 1999; Myers, 1988) or are biodiversity niches (Hanson et al., 2020). Analyses of hotspots have revealed that 35% of vertebrates and 44% of vascular plants are confined to one of 25 hotspots that cover only 1.4% of the Earth's land surface (Myers et al., 2000). Hotspots have also been used to identify priority areas for the conservation of threatened species (Mittermeier et al., 1998; Prendergast et al., 1993; Pressey et al., 1993; Williams et al., 1996). There is broad consensus that focusing conservation efforts on biodiversity hotspots would greatly assist in stemming the mass extinctions of the Anthropocene (Dinerstein et al., 2019; Myers et al., 2000). However, given that 15% of species on the IUCN Red List are classified as data deficient (IUCN, 2018), the validity of using hotspots as focal areas for conserving threatened species may be flawed. Clarifying the actual status of these data deficient species is urgent because hotspots are being rapidly eroded (de Lima et al., 2020), and if hotspots of data deficient species are not consistent with those of species richness, endemism, or threatened species, then our current understanding of hotspots will be likely biased and unrepresentative. This scenario may lead to inaccurate and inefficient prioritization of protected areas and conservation actions.

If data deficient species are distributed in a manner similar to that of better‐studied species and face similar levels of endemism and threats, then hotspots of data deficient species would coincide with hotspots of biodiversity more broadly. This assumption stems from our hypothesis that areas rich in biodiversity, with high concentrations of endemic species and facing significant threats, would also exhibit higher levels of data deficiency across species. Therefore, regions identified as hotspots for biodiversity conservation would likely overlap with regions characterized by a higher prevalence of data deficient species.

Here, we aimed to test whether data deficient species exhibit similar hotspot patterns as biodiversity hotspots (in terms of both common and threatened species) more generally. We used a multitaxon, global analysis rather than a single‐taxon, regionally focused analysis to provide a reproducible screening tool to support global survey prioritization and protected area gap analyses focused on data deficiency hotspots. We also assessed whether data deficient species richness reflects overall species richness, as we predicted similar biogeographic patterns for non‐data deficient species and data deficient species richness (i.e., productivity‐driven patterns of higher species richness closer to the equator than the poles, and evidence of elevational gradients with higher species richness at lower altitudes than at higher altitudes) (Gaston, 2000; Hawkins & Felizola Diniz‐Filho, 2004). Alternatively, socioeconomic factors affect research effort globally and might drive the distribution of data deficient species. Understanding data deficient species distributions is fundamental to prioritizing protected area creation, research, and management because it is likely data deficient species face substantial threats (Borgelt et al., 2022); additionally, there are challenges to implementing conservation actions or research when focal areas for data deficient species are poorly understood. These results can help ensure that the implementation of the 2030 Kunming–Montreal targets, which aim to protect 30% of the world's terrestrial area and 30% of the world's coastal and marine area, accounts for data deficient species.

## DATA AND METHODS

### Geoprocessing of species data

We used ArcGIS Pro 2020 (Esri, 2020) to determine the spatial distribution of species listed as data deficient on the IUCN Red List and to calculate the total number of data deficient and threatened species by country. We acknowledge that assigning a species to an IUCN Red List category is a simplistic approach as the list is infrequently updated (the goal is to reevaluate every species’ extinction risk at least every 10 years) and varies by assessors and regions, particularly in terms of when a species is categorized as data deficient; thus, the Red List is susceptible to errors and biases.

Spatial distribution data as vector polygons for all data deficient species across the world were obtained from the IUCN (https://www.iucnredlist.org/resources/spatial‐data‐download). At the time of access (30/7/2021), polygon (region) data were available for amphibians, chondrichthyans (sharks and rays), freshwater fish, marine fish, marine mammals, reptiles, and terrestrial mammals. Avian spatial distributions were obtained from Birdlife International (http://datazone.birdlife.org/species/requestdis) and joined to the Red List data using ArcGIS Pro. Although the spatial distribution of data deficient species may exhibit high degrees of uncertainty due to the lack of knowledge on them, we believe that areas where data deficient species are known to occur are most likely to be a starting place for investigations into these species. Furthermore, this uncertainty is likely to have limited impact on studies at a global scale such as this one. All spatial data were reprojected into the Equal Earth coordinate system (50 km^2^ resolution; Behrman, WKID: 54017) to ensure consistency. Hotspots were identified based on the spatial area of overlap among species distribution polygons obtained from the IUCN Red List. We used ArcGIS Pro's count overlapping polygons tool to delineate regions with a high density of overlapping polygons as hotspots. This tool allowed us to preserve the distinct ranges of individual taxa while quantifying the degree of overlap among species ranges at specific locations. The analysis was conducted for each taxonomic group, including amphibians, birds, mammals, reptiles, and fish, to highlight differences in spatial patterns of data deficiency across taxa. No specific threshold was used to define a hotspot due to each taxa having very different counts; rather, a visual interpretation of density patterns was employed to identify regions of interest. This approach ensured flexibility and allowed for the identification of biologically meaningful clusters without imposing arbitrary cutoffs that might obscure important trends.

To evaluate our project hypothesis, we overlaid the hotspots of data deficient species with hotspots of total species richness and threatened species for a combination of terrestrial species (amphibians, birds, reptiles, and land mammals). Raster data on total species richness and threatened species for amphibians, birds, mammals, and reptiles were accessed from the IUCN Red List spatial database at 10 km resolution (https://www.iucnredlist.org/resources/other‐spatial‐downloads#SR_2023). We converted the vector data of data deficient species (amphibians, birds, reptiles, and land mammals) to the raster format within Arc GIS Pro to conduct spatial overlays and statistical comparisons. Prior to analysis, datasets were resampled to 25 km resolution to ensure consistency in spatial analysis and assess the degree of overlap and correlation between these hotspot types.

To further analyze the spatial patterns of data deficiency, we calculated the ratios of data deficient species to both threatened species and total species richness by calculating the relative proportion of data deficient species in each region. This analysis highlighted areas where data deficiencies may be disproportionately affecting our understanding of threatened species distributions or species richness. These ratios were visualized using ArcGIS Pro to directly compare data deficiency across regions with varying biodiversity levels.

Finally, data deficient species hotspot maps were compared with independent datasets on the biodiversity hotspots originally defined by Myers et al. (2000). This comparison aimed to assess whether regions identified as data deficiency hotspots aligned with globally recognized biodiversity hotspots. The updated Myers et al. (2000) hotspots shapefile was downloaded from https://zenodo.org/records/3261807#.X8_iaNhKg2y (Hoffman et al., 2016).

To assess the potential drivers of data deficiency, we calculated the spatial distribution of species within each country boundary from the count polygon dataset. To calculate these values, we built and ran a workflow for each taxonomic group using ModelBuilder in ArcGIS Pro. The final global dataset containing all data deficient species was collated in R (R Core Development Team, 2008). The ArcGIS Pro geoprocessing tool dissolve was used to create multipart features to account for the fact that some species’ spatial distributions consisted of multiple polygons. World country boundaries were downloaded from https://www.igismap.com/download‐world‐shapefile‐free‐country‐borders‐continents/. For the species distribution data, the world boundary layer was reprojected into the Equal Earth coordinate system (Behrman, WKID: 54017).

To determine the spatial distribution of species within each country's boundary, the ArcGIS Pro geoprocessing tool overlay was used to map the intersection between each species polygon and each country's boundaries. The ArcGIS Pro tool summarize within was then used to calculate the total number of species within the borders of each country. A field titled taxonomic group (e.g., amphibian) was then added before merging the layers for all taxonomic groups into one file.

### Sociodemographic and geographic predictors of data deficiency

The financial contributions of countries through funding for domestic and international conservation efforts were assessed using data from Waldron et al. (2013), which includes both domestic (within‐country) spending and donations contributed from other countries, and these contributions were summed at the country level for the purpose of the present study. We expected such funding to have a significant bearing on the knowledge about a species’ status in a country. We hypothesized that more money spent on conservation was likely to flow to poorly known or data deficient species than to well‐known species (but see Garnett et al., 2003), and wealthier countries were more likely to have money available to invest in these species. Another factor affecting a country's ability to fund conservation is political will (Jones et al., 2025). For example, land clearing through agriculture is a major threat to biodiversity, and we hypothesized that countries with agriculture‐based economies were likely to have less political will and thus money available to invest in researching data deficient species.

In contrast, we also hypothesized that longer periods of compulsory education would likely result in a more educated society with more public interest and political pressure to study data deficient species. We assumed that larger countries in similar parts of the world would have greater species richness and therefore more data deficient species if the distribution of the two is related. Furthermore, human population density and the rate of population growth would likely affect the number of data deficient species in a country because more people are available to detect a species. We included continent as a variable on the assumption that some continents may have a higher proportion of data deficient species than other continents based on the heterogeneity in species richness, endemism, population density, and wealth among them. Data for the year 2020 were obtained from the World Bank World Development Indicators (https://databank.worldbank.org/source/world‐development‐indicators#, accessed 05/12/2022). The mean elevation of each country (CIA, 2024) and the latitude centroid of the center of the country were used to test whether similar environmental productivity factors that affect species richness (Gaston, 2000; Hawkins & Felizola Diniz‐Filho, 2004) also affect data deficient species. Countries for which data were incomplete were excluded from the analysis; however, for 11 countries, only years of compulsory education were missing, so these missing values were sourced by searching for this information on the Internet. The final dataset included a total of 161 countries.

### Data analysis

The total number of data deficient species was modeled using a generalized linear mixed model (GLMM) with a Poisson error distribution and log link function using the R package spaMM version 4.4.0 (Rousset, 2023). The model included a spatial term with a Matern correlation structure to account for spatial autocorrelation between locations with model comparisons using marginal Akaike Information Criteria (mAICs) and variographic analysis. Distances between country centroids were measured in great circle (orthodromic) values. There was an extensive model development process focused on identifying the nature of relationships, ranking the size of all potential effects, and determining their statistical significance. The process is not described here, but the details and associated explanatory notes are available via the R code files available in the repository noted below. The model included the following predictor variables, some of which were log_10_‐transformed to correct for right skew or large orders of magnitude: mean elevation (CIA, 2024), mean latitude of the centroid of each country's largest landmass (i.e., excluding small outlying areas/islands), total amount of funding allocated to conservation (log_10_‐transformed after adding 1), gross domestic product (GDP) (log_10_‐transformed), percentage of a country covered by agriculture, number of years of compulsory education, national land area in square kilometers (log_10_‐transformed), population density of a country (log_10_‐transformed), annual population growth rate, each country's continents, and species richness. Collinearity among the predictor variables was assessed and considered unlikely to impact the significance of model effects. The final model included all potential explanatory variables with quadratic terms to account for nonlinear relationships for land area, latitude, and species richness. To identify significant predictors of total counts, the full model was compared to models with single‐term deletions using likelihood ratio tests implemented via the drop1 function from the R package stats, version 4.3.1 (R Core Team, 2008). Effects for statistically significant factors were presented graphically as predictor effects plots to provide a way to evaluate the relative size of effects (Fox & Weisberg, 2018). Model assumptions were examined using a residual‐predicted value plot and histograms, and normal quantile plots were used for the distribution of the random effects and residuals. All 11 variables in the model were used to calculate the expected number of data deficient species for each country (termed the fixed‐effect prediction). The fixed‐effect term that was used to quantify the amount of explainable variation due to known variables used all the explanatory variables irrespective of their statistical significance. The remaining unexplained variation was partitioned into two components: a random effect, based on the spatial associations between countries, and a residual. The fixed effect, random effect, and residual values summed to the observed number of data deficient species in each country (see Appendix S1). To identify significant predictors, the full model was compared with single term deletion models using likelihood ratio tests. Model statistics for the Matern random effects term are the following: nu (ν) = 0.371; rho (ρ) > 0.001; and variance = 0.112. Statistical significance was set at the 0.05 level. The data and details on all analyses are available via the R code as well as commentary in the analysis output (html) file available in the repository (https://doi.org/10.71862/7mx5‐ep96).

## RESULTS

**FIGURE 1:**
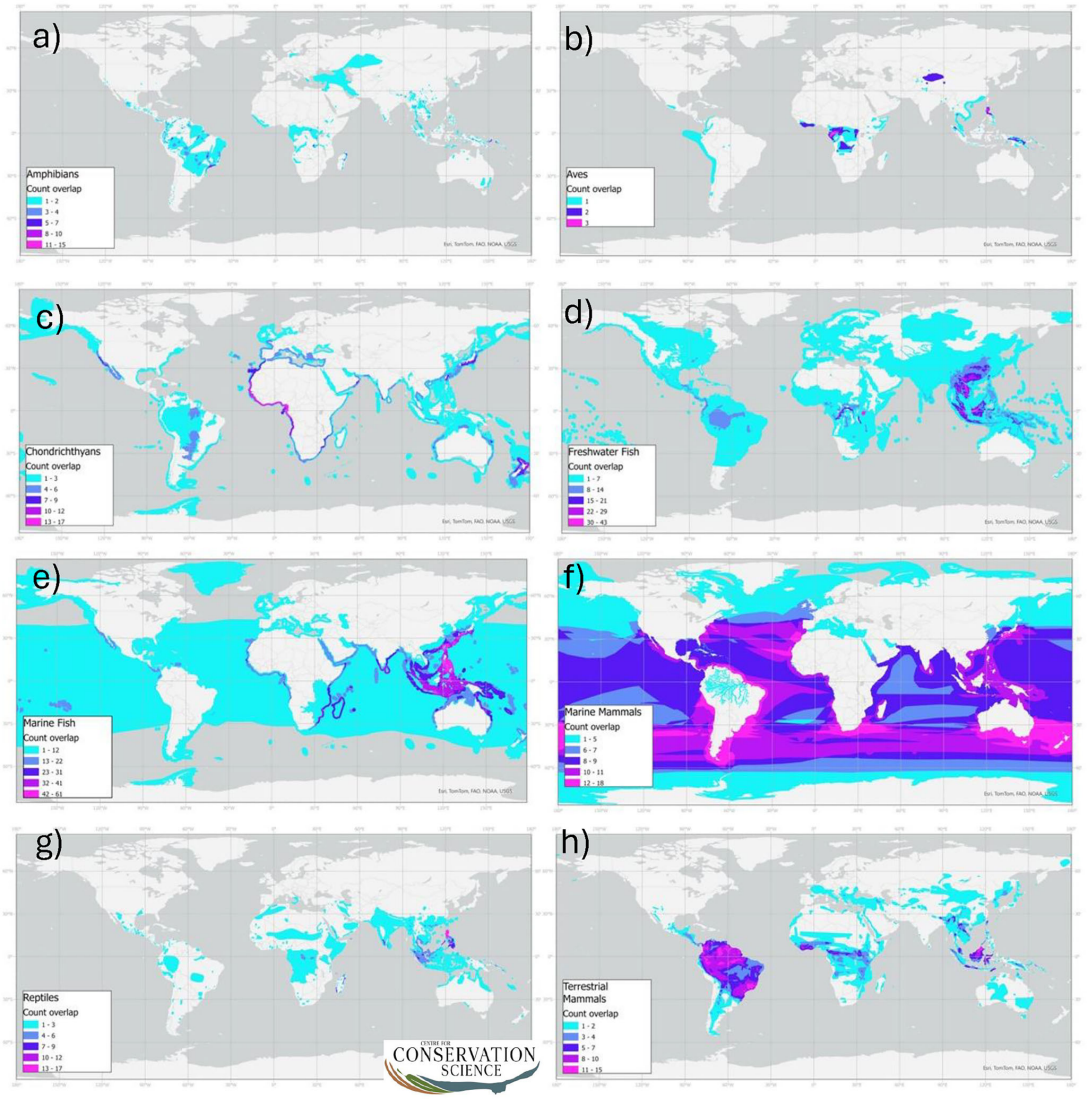
Global map of the distribution of data deficient species based on the International Union for Conservation of Nature Red List for (a) amphibians; (b) birds; (c) sharks and rays; (d) freshwater fish; (e) marine fish; (f) marine mammals; (g) herpetofauna; and (h) terrestrial mammals.

Hotspots of data deficient amphibians were concentrated around the tropical Andean forests of Colombia, Peru, and Ecuador; throughout Brazil (excluding large parts of the Cerrado and Caatinga); and in the New Guinea Highlands (Figure 1a). The tropical forests of the Congo River basin, Papua New Guinea, China's Xinjiang province, and the Philippines were hotspots for avian data deficiency (Figure 1b). Data deficient chondrichthyans were concentrated in the tropical seas, particularly along the western coast of Africa, East Asia (Philippines, Japan, and China), and the tropical Americas, as well as around New Zealand (Figure 1c). Indochina and the Congo River in Africa were the primary hotspots for data deficient freshwater fish (Figure 1d), whereas the Indo‐Pacific was the primary hotspot for data deficient marine fish (Figure 1e). Data deficient marine mammals were more widespread and occurred in a band around the temperate southern oceans, South America, Southeast Asia, and Northwest Africa (Figure 1f). Data deficient herpetofauna were concentrated around tropical Asia (Figure 1g), whereas tropical South America, Indonesia (particularly Kalimantan and Sulawesi), and Central Africa were the hotspots for data deficient terrestrial mammals (Figure 1h).

**FIGURE 2:**
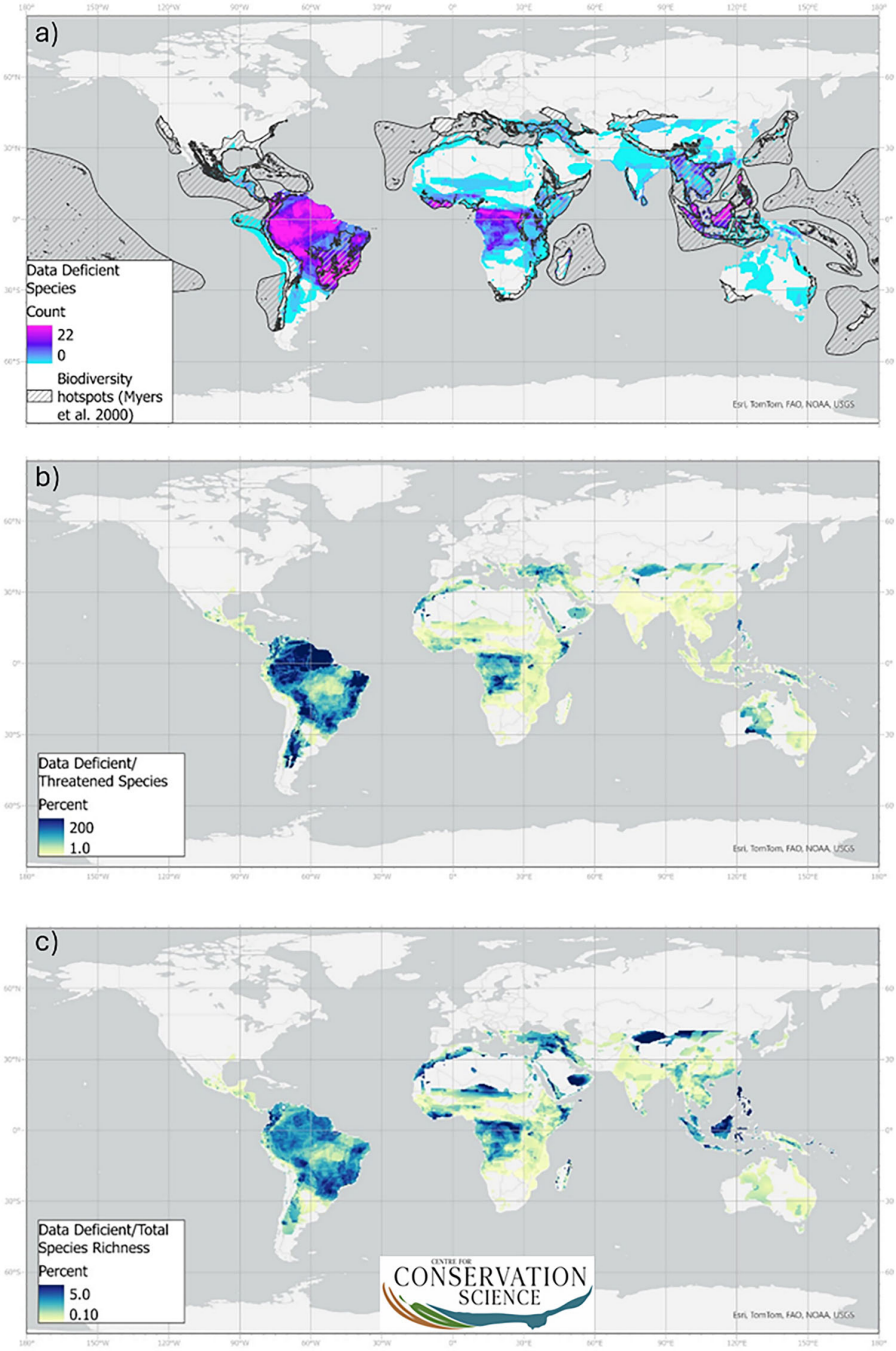
Global map of (a) the location of data deficiency hotspots and the degree of overlap between hotspots where data deficient species occur in comparison to (b) threatened species and (c) all species (this ratio is multiplied by 100 to express as a percentage). The Hoffman et al. (2016) update of the Myers et al. (2000) hotspots are plotted in panel (a).

Visual observations of hotspots of data deficiency illustrated they these areas did not simply reflect areas where threatened species occurred or where species richness was highest (Figure 2). Data deficient species were concentrated around tropical South America, Africa, and Southeast Asia (Figure 2a). This broad pattern remained when all species were combined; however, there were more data deficient species in relation to the total number of threatened species in South America, central Australia, the Congo Basin of Africa, the New Guinea Highlands, the Horn of Africa, the Balkans, and the Philippines (Figure 2b). Conversely, East Africa, eastern Australia, South and Southeast Asia, and Central America had relatively fewer data deficient species than threatened species (Figure 2b). There was a greater proportion of data deficient species in relation to total species richness in South America north of 30°S, the northern and eastern Mediterranean and the Baltic Seas, parts of the Arabian Peninsula, Indonesia, and western China (Figure 2c). There were fewer data deficient species in relation to species richness in North America, Eurasia north of 45°N, southern and Saharan Africa, New Zealand, and Australia (Figure 2c). Indonesia had the most data deficient species, followed by several other countries in Asia (China, Vietnam, and Malaysia) and Brazil in South America.

**FIGURE 3:**
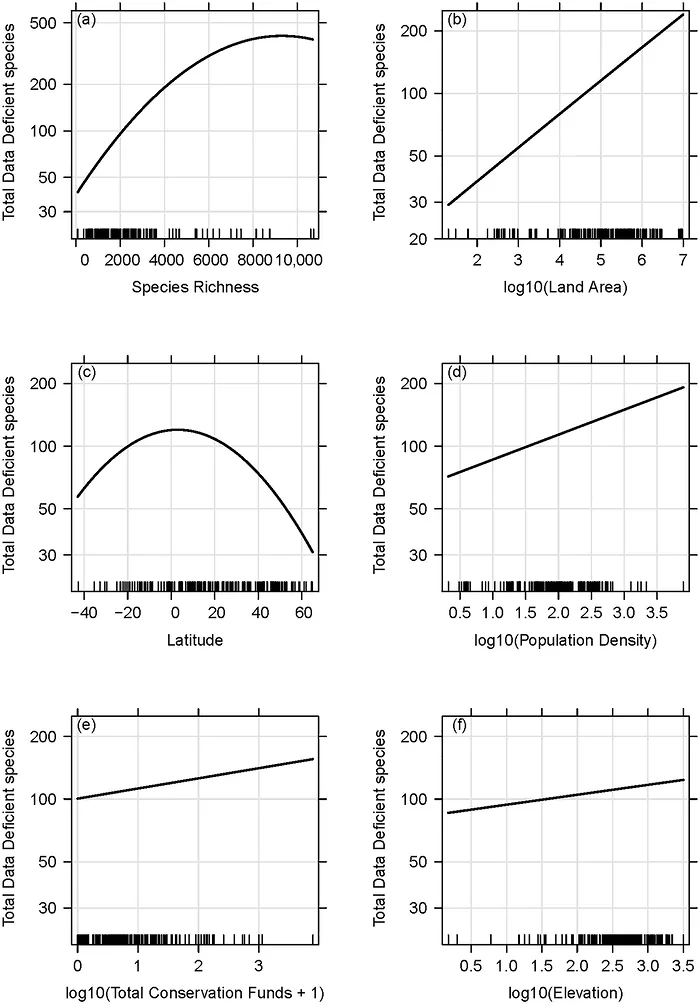
Fitted relationships between the important sociodemographic variables explaining the number of data deficient species based on the outcome of a generalized linear mixed model. These are marginal fixed effect estimates adjusted to constant values for all other variables in the model.

In addition to the visual hotspot assessment, a model‐based assessment was conducted where an adjustment was first carried out using factors that were possibly associated with data deficient species. Of the 11 factors examined, six achieved statistical significance (Table 1; Figure 3). Species richness was the strongest positive predictor of data deficient species, predicting the largest range of 40–410 data deficient species (Figure 3a), while land area was the next strongest predictor, projecting 30–240 data deficient species (Figure 3b). The curved relationship for latitude peaked at the equator, with expected numbers of data deficient species being lower closer to the poles (Figure 3c). The remaining three variables—population density, conservation funds, and elevation—had weaker relationships (Figure 3d–f). There were no associations between data deficient species and continents, percentage of agricultural land, years of education, population growth, or GDP.

**TABLE 1:** Outputs from a general linear mixed model examining the socioeconomic and demographic predictors of data deficient species based on all taxa assessed in the International Union for Conservation of Nature Red List using country‐level total counts.

Model term	Estimate	Conditional *SE*	*χ* ^2^	df	*p* value
Intercept	1.588	0.483			
log_10_(population density—people/km^2^)	0.276	0.097	7.89	1	0.005
Population growth (×10^4^)	0.84	378	0.00	1	1.00
log_10_(GDP $/10^7^)	−0.0947	0.076	1.52	1	0.22
Agricultural land (%)	−0.00251	0.0014	3.19	1	0.07
Education (years)	−0.0180	0.010	3.20	1	0.07
log_10_(land area—km^2^)	0.379	0.162	20.72	2	<0.001
log_10_(land area—km^2^)^2^	−0.00101	0.018			
Latitude (×10^4^)	20.7	32.5	14.29	2	0.001
Latitude^2^ (×10^4^)	−3.51	0.92			
log_10_(elevation)	0.110	0.053	4.22	1	0.04
log_10_(total conservation funds [$] + 1)^a^	0.112	0.045	6.09	1	0.01
Continent^b^			1.11	4	0.89
Oceania	−0.144	0.220			
Americas	−0.0619	0.190			
Asia	0.0573	0.173			
Europe	0.133	0.213			
Species richness (×10^4^)	5.09	0.543	93.347	2	<0.001
Species richness^2^ (×10^8^)	−2.73	0.470			

^a^ Total conservation funds are the sum of domestic and aid funding in Waldron et al. (2013).

^b^ The reference group for Continent is Africa.

**FIGURE 4:**
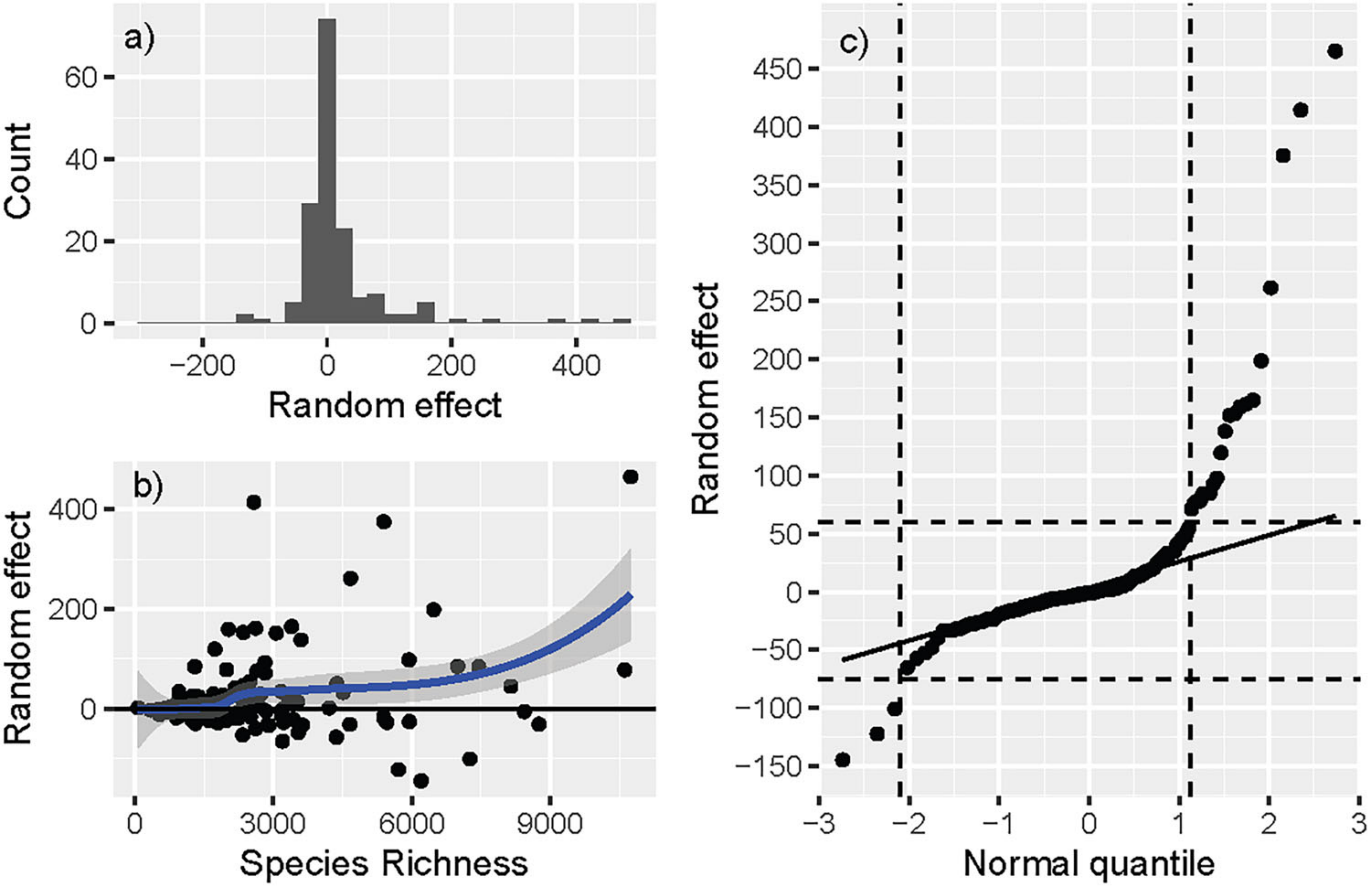
Distribution of random effects from the spatial component of the generalized linear mixed model on the response scale, that is, as numbers of data deficient species: (a) histogram; (b) random effects against species richness; and (c) a normal quantile plot.

The model‐based random effect term provided an alternative approach to hotspot assessment. The differences between the estimates of observed and fixed effects for data deficient species were dominated by the random effect term, as the residuals were small; thus, most of the unexplained variation was captured by the random effect estimates with a distribution centered on zero but with several outlying observations evident on both sides of the distribution (Figure 4a). The relationship of the random effects suggested that as species richness increased, the random effects tended to increase as well (Figure 4b). Finally, a normal quantile plot with two vertical dotted lines was used to divide the distribution into two sections (Figure 4c). First, the inner portion with the smaller random effect estimates, between the *x*‐axis quantiles −2.1 and +1.12, had an approximately normal distribution. Random effects beyond the two vertical reference lines diverged much more from the normal distribution and could be considered outliers relative to the central section. In the central portion, the random effect values ranged from −75 to + 60; however, outside this range, much larger values were evident (−100 to −150 on the negative side and up to 470 on the positive side). An equivalent version of these plots showed that the distribution of the data was largely unaffected by nonconstant variance, and the data were likely to be more normally distributed than their distribution on the response scale (https://doi.org/10.71862/7mx5‐ep96). The deviations in the outlying countries were similar, although not appearing as extreme as those in the plot.

Countries with positive outlying random effect estimates were proposed as model‐derived data deficient hotspots (Appendix S2). There were 24 outlier countries, and 21 of these countries had higher than expected numbers of data deficient species (Table 2). First, Indonesia had a high species richness (10,747), and the model‐derived number of data deficient species (based on the fixed effect) was 745. The random effect estimate for Indonesia was 465, indicating that it has 465 more species than could be explained by all the variables used in the model. This large random effect value clearly indicated a number of data deficient species beyond what would be expected. The very small residual variability (2) illustrated it had no impact on this interpretation. Thailand also had more data deficient species than expected, albeit with much lower species richness (2582) such that the fixed effect estimate (for which species richness is a major component) was much lower at 181. Although there were fewer data deficient species in Thailand than in Indonesia, the random effect was still high at 414: observed 607_(fixed effect)_ − 181_(random effect)_ − 12_(residual)_. Finally, India was one of the three countries that exhibited fewer than expected data deficient species (Table 2). Although India had high species richness (7263), the fixed effect estimate (736) was larger than the number of deficient data species, so the random effect was negative (−101), indicating that India has more knowledge about its species than almost all other countries.

**TABLE 2:** Countries identified as having unusually high or low numbers of data deficient species as defined by the random effects estimate (these countries are at the extremes of the random effect distribution in Figure 4a and more explicitly outside the vertical quantile reference lines in Figure 4c).

Continent	Country	LON	LAT	Species richness	Data deficient species	FE^a^	RE^b^	R^c^
Asia	Indonesia	114.3	−1.0	10,747	1212	744.9	465.3	1.8
Asia	Thailand	100.8	15.7	2582	607	180.9	414.4	11.7
Asia	Vietnam	105.3	21.5	5398	763	385.4	375.3	2.3
Asia	Myanmar	96.0	21.7	4677	605	343.1	261.5	0.4
Asia	Malaysia	102.2	4.2	6477	689	493.9	198.7	−3.6
Africa	Angola	17.5	−12.3	3399	378	210.5	164.9	2.6
Africa	Gabon	11.8	−0.6	2620	300	132.8	161.5	5.8
Africa	Malawi	33.8	−13.4	2031	285	112.3	159.2	13.5
Africa	Zambia	26.3	−14.6	2354	317	157.3	153.1	6.6
Asia	Cambodia	104.6	12.7	3067	333	183.5	151.9	−2.4
Africa	Cameroon	12.3	5.1	3605	409	272.0	138.1	−1.1
Africa	Central African Republic	20.5	6.6	1738	246	119.6	119.6	6.8
Americas	Peru	−75.6	−9.3	5944	500	398.9	97.9	3.2
Asia	Brunei Darussalam	114.6	4.5	2819	209	111.4	92.4	5.2
Americas	Mexico	−102.5	24.0	6997	551	464.7	84.7	1.6
Africa	Burundi	29.9	−3.4	1301	187	85.8	84.4	16.8
Asia	China	106.5	33.4	7456	753	669.1	84.3	−0.4
Africa	Equatorial Guinea	10.5	1.6	1988	172	92.0	78.2	1.8
Americas	Brazil	−53.1	−10.8	10,615	720	639.8	77.8	2.3
Africa	Guinea	−10.9	10.4	2651	233	153.1	76.1	3.8
Asia	Singapore	103.8	1.4	2828	175	100.1	71.2	3.8
Asia	India	78.5	21.0	7263	633	735.5	−100.8	−1.7
Africa	South Africa	23.1	−30.6	5715	177	303.5	−122.4	−4.1
Asia	Philippines	122.5	11.1	6206	324	476.0	−144.7	−7.3

^a^ FE = fixed effect.

^b^ RE = random effect. The REs were obtained from the spatial component of the model after adjustment for explanatory variables using the model FE estimate.

^c^ R = the model residuals. The model estimates (FE + RE + R) sum to the number of observed data deficient species.

## DISCUSSION

This study shows that data deficient species are not distributed evenly across the world, and although the number of data deficient species follows global patterns in species richness (Gaston, 2000; Gillman et al., 2015), there are hotspots of data deficiency that do not coincide with hotspots of biodiversity or threatened species (Figure 1; Hoffman et al., 2016; Myers et al., 2000). Because of this scenario, our current understanding of the distribution of threatened species may be flawed, limiting our ability to conserve biodiversity, as many poorly known species that may be at risk of extinction do not have distributions overlapping global biodiversity hotspots. Although biodiversity hotspots typically include areas with habitat loss as well, our goal was to assess whether we could use these areas as proxies for data deficient hotspots. Our data deficiency hotspots reflected concentrated areas of data deficiency based on simple overlap counts; thus, these areas require additional survey and research efforts to understand the status of data deficient species to ensure that global threatened species assessments, and likely other hotspots, are thorough and robust. Until we have a more complete understanding of the distribution of threatened species, spatial prioritization of protected areas will be unlikely to maximize conservation success, and our ability to appropriately respond to the global biodiversity crisis will be hindered. Addressing this issue is urgent because although progress to conserve hotspot areas is occurring, the window of opportunity for expanding the global protected area network is rapidly closing (Jenkins et al., 2013).

To date, we have assumed that hotspots of known threatened species are the appropriate foci for conservation efforts based on the assumption that they reflect areas where the greatest number of threatened species occurs (Myers et al., 2000). Although the relationship between global species richness and data deficient species illustrates that this assumption is partially true, it is clear that some countries exhibit greater than expected levels of data deficiency and that a few countries exhibit lower than expected levels of data deficiency (Figure 2; Table 2). Hence, existing hotspots are based on an incomplete dataset. Furthermore, and equally concerning, evidence shows that data deficient species are disproportionately threatened with extinction (Bland & Böhm, 2016; Wotherspoon et al., 2024).

The lack of congruence between taxa that occurred in data deficiency hotspots was perhaps unsurprising because it reflects the contrasting results between hotspots of endemic plants and animals (Myers et al., 2000). More concerning was the lack of congruence between threatened and data deficient species because the use of rare species hotspots has been promoted as a method to prioritize protected areas (Davidson & Dulvy, 2017; Huang et al., 2020; Myers et al., 2000). However, Prendergast et al. (1993) found that there was little congruence between species‐rich areas of Great Britain and areas where threatened species occurred. Orme et al. (2005) showed a similar lack of correspondence for birds and considered the dissimilarity between threatened species and endemism/species richness due to human impacts. Ceballos and Ehrlich (2006) showed that there was little congruence among the distribution of species richness, endemic species, and threatened species, and concluded that this was because global species richness is driven by widespread species, whereas ecological and evolutionary processes act differently in determining the geographic ranges of widespread and range‐restricted species. Hence, protected areas prioritized using hotspot analyses relying on threatened species are based on inadequate data because data deficient species are not accurately assigned to a threat status category. If these data deficient species are ultimately incorporated into the IUCN Red List as threatened when sufficient data are obtained, then existing protected areas are likely to be incorrectly located, as surrogacy between diversity metrics cannot be assumed (Orme et al., 2005). Nonetheless, recent analyses using complex trait‐based modeling have shown a greater degree of overlap between data deficient and threatened terrestrial reptile species than had been previously reported (Bland & Böhm, 2016), and Bayesian modeling offers opportunities to reduce the impact of some of the uncertainty associated with data deficiency (Newton, 2010).

At a species level, different taxa exhibited different degrees of data deficiency (Figure 1), which is further emphasized by the global gaps in trait data for terrestrial vertebrates (Etard et al., 2020). This scenario is a cause for concern in amphibians given that 1404 species are categorized as data deficient and may be at risk of extinction without appropriately targeted conservation effort (González‐del‐Pliego et al., 2019). Collectively, amphibians represent the most highly threatened class of vertebrates (Catenazzi, 2015). Unfortunately, the variety of threats to amphibians around the world (IUCN, 2020; but see Re:wild et al., 2023) makes it hard to extrapolate the threats from well‐known species to data deficient species. Without biological and ecological data, many of the world's amphibians are in danger of becoming extinct before we can even identify the causes of their decline (Catenazzi, 2015).

In terms of the factors affecting data deficiency, a country's species richness, land area, and proximity to the equator (latitude reflects the importance of productivity in driving species richness; Table 2) had the greatest effects. Population density, the conservation funds a country receives, and elevation had smaller effects on data deficiency, whereas the other covariates had no effect. Counterintuitively, population density and conservation funding showed a positive relationship with data deficiency, rejecting our hypothesis that more people in a country would enable a greater understanding of poorly known species and suggesting that either funds are not helping address species knowledge gaps or those funds are going toward species discovery and description given that the majority of species remain to be discovered and described (Hortal et al., 2015). Although increased elevation tends to reduce species richness (Davidson & Dulvy, 2017), data deficient species increased with elevation (Figure 3), suggesting that the challenges of working in higher altitude areas are inhibiting our ability to understand the biodiversity that lives there.

We assumed that higher levels of education would imply that more researchers were studying data deficient species; however, this hypothesis was also not supported. There could be many reasons for this result, but the publishing focus of academia on high‐impact research may be an issue given that much of the information needed for data deficient species could be seen as natural history research, which is poorly supported (Nanglu et al., 2023; Tewksbury et al., 2014) despite being critically important in assessing species status (Drew, 2025).

Several countries had more or less than the expected number of data deficient species (Table 2). Indonesia, Thailand, Vietnam, and Myanmar had more than the expected number of data deficient species, and these countries tend to support high biodiversity coupled with intermediate levels of conservation spending and population density. Conversely, countries with fewer than expected data deficient species, such as India and South Africa, have had a long European colonial history of hunting, which often has led to an increase in formalized or documented natural history understanding (MacKenzie, 1997).

There are related areas of study that require more research. Because of the counterintuitive positive relationship between data deficiency and population density and conservation funding, gaining a deeper understanding of the drivers of data deficiency among wealthier and more populated countries seems an important avenue of research. This relationship may also be attributable to the biases associated with the collection of socioeconomic and biodiversity data (Chapman et al., 2024; Ellis‐Soto et al., 2024); however, previous studies have used these types of data (e.g., Waldron et al., 2013), and we believe their use is valid here. Similarly, more directed research by conservation scientists, supported by managers and funding agents, may be necessary to specifically target data deficient species.

It is also important to recognize that data deficiency does not mean species are receiving no conservation attention or protection, in the same way that a threatened status does indicate that a species is receiving conservation. Nonetheless, improving our understanding of data deficient species will optimize our ability to conserve a broader suite of biodiversity given constraints on conservation funding. Species that have not been assessed for the IUCN Red List remain a source of potential bias for hotspot analyses, and increasing the number of species across all taxa that have had their extinction risk assessed needs to remain a priority. Hence, undescribed species similarly remain problematic and should be prioritized.

It has been long recognized that hotspots need to be revised as more robust data become available (Ceballos & Ehrlich, 2006; Possingham & Wilson, 2005) or when taxonomic revisions occur; however, our study identifies where targeted studies should be conducted to improve the quality of data for determining hotspot locations. It is imperative that this effort begins soon, before we lose more species about which we know very little. Indeed, directing research efforts toward as few as 6199 of the world's protected areas (or 0.36% of the world's terrestrial surface) would likely assist in gathering information on 42% of data deficient species (Nori et al., 2020). For amphibians, a focus on 0.4% of the world's terrestrial area would yield information on more than 80% of data deficient species (Nori et al., 2018).

Given the high levels of data deficiency in the Global South (Figure 2) and a recognition of the need to decolonize conservation (Domínguez & Luoma, 2020), scientists from the Global South should be supported in targeting research in data deficiency hotspots. This effort could be implemented by attracting scientists to conduct research in these areas and training more local scientists; however, remote‐controlled scientific practices must be avoided by involving more marginalized researchers, such as those from the Global South, early in the process and recognizing local knowledge, fair authorship, capacity building, and improved funding (Dáttilo & Rivera‐Núñez, 2025). Identifying gaps in our knowledge is fundamental to efficiently and effectively averting the biodiversity crisis (Christie et al., 2021), and working with local scientists to focus research on data deficiency hotspots will assist in addressing these gaps.

## Supporting information




**Supplementary Table S1**. Random effects for all countries, in descending order of size.
**Supplementary Table S2**. Top ten countries housing Data Deficient species a) overall and b) by area based on all taxa assessed in the IUCN Red List.

## Data Availability

The data and R code used in these analyses are available at https://doi.org/10.71862/7mx5‐ep96.

## References

[cobi70341-bib-0001] Bland, L. M. , & Böhm, M. (2016). Overcoming data deficiency in reptiles. Biological Conservation, 204, 16–22.

[cobi70341-bib-0002] Bland, L. M. , Collen, B. , Orme, C. D. L. , & Bielby, J. (2015). Predicting the conservation status of data‐deficient species. Conservation Biology, 29, 250–259. 10.1111/cobi.12372 25124400

[cobi70341-bib-0003] Borgelt, J. , Dorber, M. , Høiberg, M. A. , & Verones, F. (2022). More than half of data deficient species predicted to be threatened by extinction. Communications Biology, 5, Article 679. 10.1038/s42003-022-03638-9 PMC9352662 35927327

[cobi70341-bib-0004] Catenazzi, A. (2015). State of the world's amphibians. Annual Review of Environment and Resources, 40, 91–119.

[cobi70341-bib-0005] Ceballos, G. , & Ehrlich, P. R. (2006). Global mammal distributions, biodiversity hotspots, and conservation. Proceedings of the National Academy of Sciences, 103, 19374–19379. 10.1073/pnas.0609334103 PMC1698439 17164331

[cobi70341-bib-0006] Central Intelligence Agency (CIA) . (2024). Elevation. In The world factbook. Simon & Schuster.

[cobi70341-bib-0007] Chapman, M. , Goldstein, B. R. , Schell, C. J. , Brashares, J. S. , Carter, N. H. , Ellis‐Soto, D. , Faxon, H. O. , Goldstein, J. E. , Halpern, B. S. , Longdon, J. , Norman, K. E. A. , O'Rourke, D. , Scoville, C. , Xu, L. , & Boettiger, C. (2024). Biodiversity monitoring for a just planetary future. Science, 383, 34–36. 10.1126/science.adh8874 38175872

[cobi70341-bib-0008] Christie, A. P. , Amano, T. , Martin, P. A. , Petrovan, S. O. , Shackelford, G. E. , Simmons, B. I. , Smith, R. K. , Williams, D. R. , Wordley, C. F. , & Sutherland, W. J. (2021). The challenge of biased evidence in conservation. Conservation Biology, 35, 249–262. 10.1111/cobi.13577 32583521

[cobi70341-bib-0009] Dáttilo, W. , & Rivera‐Núñez, T. (2025). Remote‐control science in ecology: A hidden face of scientific neocolonialism. Ecology Letters, 28, Article e70227. 10.1111/ele.70227 41035183

[cobi70341-bib-0010] Davidson, L. N. K. , & Dulvy, N. K. (2017). Global marine protected areas to prevent extinctions. Nature Ecology & Evolution, 1, Article 0040. 10.1038/s41559-016-0040 28812606

[cobi70341-bib-0011] de Lima, R. A. , Oliveira, A. A. , Pitta, G. R. , de Gasper, A. L. , Vibrans, A. C. , Chave, J. , Ter Steege, H. , & Prado, P. I. (2020). The erosion of biodiversity and biomass in the Atlantic Forest biodiversity hotspot. Nature Communications, 11, Article 6347. 10.1038/s41467-020-20217-w PMC7733445 33311511

[cobi70341-bib-0012] Dinerstein, E. , Vynne, C. , Sala, E. , Joshi, A. R. , Fernando, S. , Lovejoy, T. E. , Mayorga, J. , Olson, D. , Asner, G. P. , Baillie, J. E. M. , Burgess, N. D. , Burkart, K. , Noss, R. F. , Zhang, Y. P. , Baccini, A. , Birch, T. , Hahn, N. , Joppa, L. N. , & Wikramanayake, E. (2019). A global deal for nature: Guiding principles, milestones, and targets. Science Advances, 5, Article eaaw2869. 10.1126/sciadv.aaw2869 PMC6474764 31016243

[cobi70341-bib-0013] Domínguez, L. , & Luoma, C. (2020). Decolonising conservation policy: How colonial land and conservation ideologies persist and perpetuate indigenous injustices at the expense of the environment. Land, 9, Article 65.

[cobi70341-bib-0014] Drew, L. (2025). Want to get a species protected? Publish in a small, niche journal. Nature. 10.1038/d41586-025-00144-w 39881506

[cobi70341-bib-0015] Ellis‐Soto, D. , Chapman, M. , & Koltz, A. M. (2024). Addressing data disparities is critical for biodiversity assessments. Trends in Ecology & Evolution, 39, 1066–1069. 10.1016/j.tree.2024.10.005 39603909

[cobi70341-bib-0055] Esri . (2020). World Topographic Map [basemap]. ArcGIS Online. February 19, 2020. Available: Esri ArcGIS Online.

[cobi70341-bib-0016] Etard, A. , Morrill, S. , & Newbold, T. (2020). Global gaps in trait data for terrestrial vertebrates. Global Ecology and Biogeography, 29, 2143–2158.

[cobi70341-bib-0017] Fox, J. , & Weisberg, S. (2018). Visualizing fit and lack of fit in complex regression models with predictor effect plots and partial residuals. Journal of Statistical Software, 87, 1–27.

[cobi70341-bib-0018] Garnett, S. , Crowley, G. , & Balmford, A. (2003). The costs and effectiveness of funding the conservation of Australian threatened birds. Bioscience, 53, Article 658.

[cobi70341-bib-0019] Gaston, K. J. (2000). Global patterns in biodiversity. Nature, 405, 220–227. 10.1038/35012228 10821282

[cobi70341-bib-0020] Gillman, L. N. , Wright, S. D. , Cusens, J. , McBride, P. D. , Malhi, Y. , & Whittaker, R. J. (2015). Latitude, productivity and species richness. Global Ecology and Biogeography, 24, 107–117.

[cobi70341-bib-0021] Ginsberg, J. (1999). Global conservation priorities. Conservation Biology, 13, 5.

[cobi70341-bib-0022] González‐del‐Pliego, P. , Freckleton, R. P. , Edwards, D. P. , Koo, M. S. , Scheffers, B. R. , Pyron, R. A. , & Jetz, W. (2019). Phylogenetic and trait‐based prediction of extinction risk for data‐deficient amphibians. Current Biology, 29, 1557.e3–1563.e3. 10.1016/j.cub.2019.04.005 31063716

[cobi70341-bib-0023] Hanson, J. O. , Rhodes, J. R. , Butchart, S. H. M. , Buchanan, G. M. , Rondinini, C. , Ficetola, G. F. , & Fuller, R. A. (2020). Global conservation of species’ niches. Nature, 580, 232–234. 10.1038/s41586-020-2138-7 32269340

[cobi70341-bib-0024] Hawkins, B. A. , & Felizola Diniz‐Filho, J. A. (2004). ‘Latitude’ and geographic patterns in species richness. Ecography, 27, 268–272.

[cobi70341-bib-0025] Hoffman, M. , Koenig, K. , Bunting, G. , Costanza, J. , & Williams, K. J. (2016). Biodiversity hotspots (Version 2016.1) [Data set]. Zenodo. 10.5281/zenodo.3261807

[cobi70341-bib-0026] Hortal, J. , de Bello, F. , Diniz‐Filho, J. A. F. , Lewinsohn, T. M. , Lobo, J. M. , & Ladle, R. J. (2015). Seven shortfalls that beset large‐scale knowledge of biodiversity. Annual Review of Ecology, Evolution, and Systematics, 46, 523–549.

[cobi70341-bib-0027] Huang, Z. , Bai, Y. , Alatalo, J. M. , & Yang, Z. (2020). Mapping biodiversity conservation priorities for protected areas: A case study in Xishuangbanna Tropical Area, China. Biological Conservation, 249, Article 108741.

[cobi70341-bib-0028] International Union for Conservation of Nature (IUCN) . (2012). IUCN Red List Categories and Criteria (Version 3.1). Author.

[cobi70341-bib-0029] International Union for Conservation of Nature (IUCN) . (2018). 2018 Red List of Threatened Species . www.redlist.org

[cobi70341-bib-0030] International Union for Conservation of Nature (IUCN) . (2020). Summary Statistics 2020 . https://www.iucnredlist.org/resources/summary‐statistics

[cobi70341-bib-0053] Jones, J. , Griffin, A. S. , Agbola, F. W. , & Hayward, M. W. (2025). Role of national regime ideology for predicting biodiversity outcomes. Conservation Biology, 39, e14314. 10.1111/cobi.14314 PMC11780220 39105482

[cobi70341-bib-0031] Jenkins, C. N. , Pimm, S. L. , & Joppa, L. N. (2013). Global patterns of terrestrial vertebrate diversity and conservation. Proceedings of the National Academy of Sciences, 110, E2602–E2610. 10.1073/pnas.1302251110 PMC3710798 23803854

[cobi70341-bib-0054] Mace, G. M. , & Lande, R. (1991). Assessing extinction threats: towards a re‐evaluation of IUCN threatened species categories. Conservation Biology, 5, 148–157.

[cobi70341-bib-0032] MacKenzie, J. M. (1997). The empire of nature: Hunting, conservation, and British imperialism. Manchester University Press.

[cobi70341-bib-0033] Mittermeier, R. A. , Myers, N. , Thomsen, J. B. , Da Fonseca, G. A. B. , & Olivieri, S. (1998). Biodiversity hotspots and major tropical wilderness areas: Approaches to setting conservation priorities. Conservation Biology, 12, 516–520.

[cobi70341-bib-0034] Myers, N. (1988). Threatened biotas: “Hot spots” in tropical forests. The Environmentalist, 8, 187–208. 10.1007/BF02240252 12322582

[cobi70341-bib-0035] Myers, N. , Mittermeier, R. A. , Mittermeier, C. G. , da Fonseca, G. A. B. , & Kent, J. (2000). Biodiversity hotspots for conservation priorities. Nature, 403, 853–858. 10.1038/35002501 10706275

[cobi70341-bib-0036] Nanglu, K. , De Carle, D. , Cullen, T. M. , Anderson, E. B. , Arif, S. , Castañeda, R. A. , Chang, L. M. , Iwama, R. E. , Fellin, E. , Manglicmot, R. C. , Massey, M. D. , & Astudillo‐Clavijo, V. (2023). The nature of science: The fundamental role of natural history in ecology, evolution, conservation, and education. Ecology and Evolution, 13, Article e10621. 10.1002/ece3.10621 PMC10591213 37877102

[cobi70341-bib-0037] Newton, A. C. (2010). Use of a Bayesian network for Red Listing under uncertainty. Environmental Modelling & Software, 25, 15–23.

[cobi70341-bib-0038] Nori, J. , & Loyola, R. (2015). On the worrying fate of data deficient amphibians. PLoS ONE, 10, Article e0125055. 10.1371/journal.pone.0125055 PMC4428885 25965422

[cobi70341-bib-0039] Nori, J. , Loyola, R. , & Villalobos, F. (2020). Priority areas for conservation of and research focused on terrestrial vertebrates. Conservation Biology, 34, 1281–1291. 10.1111/cobi.13476 32009235

[cobi70341-bib-0040] Nori, J. , Villalobos, F. , & Loyola, R. (2018). Global priority areas for amphibian research. Journal of Biogeography, 45, 2588–2594.

[cobi70341-bib-0041] Orme, C. D. L. , Davies, R. G. , Burgess, M. , Eigenbrod, F. , Pickup, N. , Olson, V. A. , Webster, A. J. , Ding, T.‐S. , Rasmussen, P. C. , Ridgely, R. S. , Stattersfield, A. J. , Bennett, P. M. , Blackburn, T. M. , Gaston, K. J. , & Owens, I. P. F. (2005). Global hotspots of species richness are not congruent with endemism or threat. Nature, 436, 1016–1019. 10.1038/nature03850 16107848

[cobi70341-bib-0042] Possingham, H. P. , & Wilson, K. A. (2005). Turning up the heat on hotspots. Nature, 436, 919–920. 10.1038/436919a 16107821

[cobi70341-bib-0043] Prendergast, J. , Quinn, R. , Lawton, J. , Eversham, B. , & Gibbons, D. (1993). Rare species, the coincidence of diversity hotspots and conservation strategies. Nature, 365, 335–337.

[cobi70341-bib-0044] Pressey, R. , Humphries, C. , Margules, C. R. , Vane‐Wright, R. , & Williams, P. (1993). Beyond opportunism: Key principles for systematic reserve selection. Trends in Ecology & Evolution, 8, 124–128. 10.1016/0169-5347(93)90023-I 21236127

[cobi70341-bib-0045] R Core Development Team . (2008). R: A language and environment for statistical computing. R Foundation for Statistical Computing.

[cobi70341-bib-0046] Re:wild ., Synchronicity Earth ., & IUCN SSC Amphibian Specialist Group .. (2023). State of the World's Amphibians: Second Global Amphibian Assessment . Author.

[cobi70341-bib-0047] Rousset, M. F. (2023). spaMM: Mixed‐effect models, with or without spatial random effects . R package.

[cobi70341-bib-0048] Tewksbury, J. J. , Anderson, J. G. T. , Bakker, J. D. , Billo, T. J. , Dunwiddie, P. W. , Groom, M. J. , Hampton, S. E. , Herman, S. G. , Levey, D. J. , Machnicki, N. J. , Del Rio, C. M. , Power, M. E. , Rowell, K. , Salomon, A. K. , Stacey, L. , Trombulak, S. C. , & Wheeler, T. A. (2014). Natural history's place in science and society. BioScience, 64, 300–310.

[cobi70341-bib-0049] Tilker, A. , Abrams, J. F. , Nguyen, A. , Hörig, L. , Axtner, J. , Louvrier, J. , Rawson, B. M. , Nguyen, H. A. Q. , Guegan, F. , Nguyen, T. V. , Le, M. , Sollmann, R. , & Wilting, A. (2020). Identifying conservation priorities in a defaunated tropical biodiversity hotspot. Diversity and Distributions, 26, 426–440.

[cobi70341-bib-0050] Waldron, A. , Mooers, A. O. , Miller, D. C. , Nibbelink, N. , Redding, D. , Kuhn, T. S. , Roberts, J. T. , & Gittleman, J. L. (2013). Targeting global conservation funding to limit immediate biodiversity declines. Proceedings of the National Academy of Sciences, 110, 12144–12148. 10.1073/pnas.1221370110 PMC3718168 23818619

[cobi70341-bib-0051] Williams, P. , Gibbons, D. , Margules, C. , Rebelo, A. , Humphries, C. , & Pressey, R. (1996). A comparison of richness hotspots, rarity hotspots, and complementary areas for conserving diversity of British birds. Conservation Biology, 10, 155–174.

[cobi70341-bib-0052] Wotherspoon, L. , De Oliveira Caetano, G. H. , Roll, U. , Meiri, S. , Pili, A. , Tingley, R. , & Chapple, D. G. (2024). Inferring the extinction risk of Data Deficient and Not Evaluated Australian squamates. Austral Ecology, 49, Article e13485.

