# Supplementary material for: Identifying hotspots of faunal data deficiency to direct urgent research and monitoring
Source: Conserv Biol. 2026 Jun 4;40(5):e70341. doi: 10.1111/cobi.70341 (PMC13613548; doi:10.1111/cobi.70341)
Supplement: Supplementary file 1 [file COBI-40-e70341-s001.docx]

**Supplementary Table S1. Random effects for all countries, in descending order of size.**

| REGION | NAME | LON | LAT | DD_TOT | SpRich | FE | RE | R |
| --- | --- | --- | --- | --- | --- | --- | --- | --- |
| Asia | Indonesia | 114.3 | -1.0 | 1212 | 10747 | 744.9 | 465.3 | 1.8 |
| Asia | Thailand | 100.8 | 15.7 | 607 | 2582 | 180.9 | 414.4 | 11.7 |
| Asia | Vietnam | 105.3 | 21.5 | 763 | 5398 | 385.4 | 375.3 | 2.3 |
| Asia | Myanmar | 96.0 | 21.7 | 605 | 4677 | 343.1 | 261.5 | 0.4 |
| Asia | Malaysia | 102.2 | 4.2 | 689 | 6477 | 493.9 | 198.7 | -3.6 |
| Africa | Angola | 17.5 | -12.3 | 378 | 3399 | 210.5 | 164.9 | 2.6 |
| Africa | Gabon | 11.8 | -0.6 | 300 | 2620 | 132.8 | 161.5 | 5.8 |
| Africa | Malawi | 33.8 | -13.4 | 285 | 2031 | 112.3 | 159.2 | 13.5 |
| Africa | Zambia | 26.3 | -14.6 | 317 | 2354 | 157.3 | 153.1 | 6.6 |
| Asia | Cambodia | 104.6 | 12.7 | 333 | 3067 | 183.5 | 151.9 | -2.4 |
| Africa | Cameroon | 12.3 | 5.1 | 409 | 3605 | 272.0 | 138.1 | -1.1 |
| Africa | Central African Republic | 20.5 | 6.6 | 246 | 1738 | 119.6 | 119.6 | 6.8 |
| Americas | Peru | -75.6 | -9.3 | 500 | 5944 | 398.9 | 97.9 | 3.2 |
| Asia | Brunei Darussalam | 114.6 | 4.5 | 209 | 2819 | 111.4 | 92.4 | 5.2 |
| Americas | Mexico | -102.5 | 24.0 | 551 | 6997 | 464.7 | 84.7 | 1.6 |
| Africa | Burundi | 29.9 | -3.4 | 187 | 1301 | 85.8 | 84.4 | 16.8 |
| Asia | China | 106.5 | 33.4 | 753 | 7456 | 669.1 | 84.3 | -0.4 |
| Africa | Equatorial Guinea | 10.5 | 1.6 | 172 | 1988 | 92.0 | 78.2 | 1.8 |
| Americas | Brazil | -53.1 | -10.8 | 720 | 10615 | 639.8 | 77.8 | 2.3 |
| Africa | Guinea | -10.9 | 10.4 | 233 | 2651 | 153.1 | 76.1 | 3.8 |
| Asia | Singapore | 103.8 | 1.4 | 175 | 2828 | 100.1 | 71.2 | 3.8 |
| Africa | Uganda | 32.4 | 1.3 | 235 | 2503 | 176.6 | 54.7 | 3.6 |
| Americas | Panama | -80.9 | 8.4 | 259 | 4377 | 203.4 | 50.3 | 5.4 |
| Africa | Sierra Leone | -11.8 | 8.6 | 166 | 2361 | 117.8 | 47.3 | 0.9 |
| Americas | Colombia | -73.1 | 3.9 | 609 | 8148 | 562.3 | 45.0 | 1.7 |
| Asia | Bangladesh | 89.9 | 24.2 | 164 | 2174 | 121.5 | 41.3 | 1.2 |
| Africa | Togo | 1.1 | 8.8 | 143 | 2185 | 98.0 | 40.4 | 4.5 |
| Africa | Nigeria | 8.1 | 9.6 | 259 | 3174 | 225.6 | 34.6 | -1.2 |
| Asia | Kazakhstan | 67.3 | 48.2 | 80 | 958 | 37.7 | 34.0 | 8.4 |
| Africa | Benin | 2.5 | 10.5 | 161 | 2192 | 127.1 | 33.1 | 0.7 |
| Africa | Liberia | -9.7 | 6.7 | 160 | 2286 | 128.1 | 33.1 | -1.2 |
| Africa | Mozambique | 37.9 | -14.4 | 326 | 4518 | 296.1 | 32.7 | -2.8 |
| Europe | Croatia | 16.7 | 45.7 | 88 | 1707 | 54.9 | 29.4 | 3.7 |
| Africa | Ghana | -1.2 | 8.0 | 178 | 2730 | 151.1 | 29.2 | -2.4 |
| Africa | Guinea-Bissau | -14.7 | 12.1 | 113 | 1902 | 83.8 | 27.4 | 1.8 |
| Europe | Bosnia and Herzegovina | 17.8 | 44.2 | 73 | 1259 | 42.8 | 25.2 | 5.0 |
| Europe | Albania | 20.1 | 41.1 | 80 | 1389 | 50.2 | 23.8 | 6.1 |
| Americas | Canada | -109.4 | 59.1 | 88 | 2313 | 64.0 | 21.3 | 2.8 |
| Europe | Bulgaria | 25.2 | 42.8 | 71 | 1287 | 47.2 | 19.8 | 4.0 |
| Europe | Montenegro | 19.3 | 42.8 | 61 | 1387 | 40.2 | 19.1 | 1.7 |
| Americas | Argentina | -65.2 | -35.4 | 147 | 3333 | 125.3 | 18.6 | 3.1 |
| Europe | Slovenia | 14.8 | 46.1 | 58 | 954 | 32.1 | 17.6 | 8.3 |
| Oceania | New Zealand | 172.2 | -42.6 | 62 | 1669 | 41.2 | 17.4 | 3.3 |
| Europe | Ukraine | 31.4 | 49.0 | 66 | 1184 | 47.4 | 15.5 | 3.1 |
| Asia | Bhutan | 90.4 | 27.4 | 79 | 948 | 62.9 | 15.4 | 0.7 |
| Americas | Guatemala | -90.4 | 15.3 | 187 | 3528 | 171.8 | 14.0 | 1.3 |
| Africa | Senegal | -14.9 | 15.0 | 132 | 2398 | 120.7 | 13.6 | -2.3 |
| Europe | Romania | 25.0 | 45.8 | 58 | 1291 | 43.7 | 13.5 | 0.8 |
| Africa | Sao Tome and Principe | 6.6 | 0.2 | 45 | 964 | 34.3 | 13.4 | -2.7 |
| Africa | Mauritania | -10.3 | 20.3 | 107 | 1806 | 91.8 | 13.2 | 2.0 |
| Europe | Serbia | 20.8 | 44.0 | 47 | 1089 | 41.1 | 10.9 | -5.0 |
| Europe | Greece | 21.8 | 39.7 | 90 | 2579 | 82.8 | 10.2 | -3.0 |
| Americas | Bolivia | -64.7 | -16.7 | 186 | 3447 | 173.8 | 10.1 | 2.2 |
| Africa | Burkina Faso | -1.7 | 12.3 | 82 | 998 | 76.8 | 7.0 | -1.8 |
| Europe | Italy | 12.8 | 42.7 | 89 | 2547 | 84.6 | 6.5 | -2.1 |
| Europe | Austria | 14.9 | 47.7 | 46 | 1218 | 44.5 | 6.3 | -4.7 |
| Europe | Germany | 9.9 | 51.1 | 55 | 1349 | 48.3 | 5.1 | 1.7 |
| Europe | Hungary | 19.1 | 47.1 | 35 | 925 | 34.9 | 4.8 | -4.7 |
| Africa | Mali | -3.5 | 17.4 | 92 | 1155 | 87.0 | 4.8 | 0.3 |
| Oceania | Fiji | 178.0 | -17.8 | 75 | 2697 | 69.6 | 4.3 | 1.1 |
| Americas | El Salvador | -88.9 | 13.7 | 79 | 1878 | 73.1 | 3.4 | 2.6 |
| Europe | Sweden | 15.3 | 62.0 | 32 | 884 | 26.4 | 3.1 | 2.5 |
| Europe | Denmark | 9.3 | 56.1 | 28 | 865 | 23.4 | 2.9 | 1.7 |
| Asia | Azerbaijan | 47.4 | 40.4 | 45 | 740 | 36.6 | 2.4 | 6.0 |
| Africa | Rwanda | 29.9 | -2.0 | 72 | 1281 | 88.5 | 2.3 | -18.8 |
| Oceania | Marshall Islands | 169.0 | 7.6 | 33 | 1800 | 28.9 | 2.2 | 1.8 |
| Europe | San Marino | 12.5 | 43.9 | 13 | 77 | 11.4 | 1.8 | -0.2 |
| Oceania | Vanuatu | 166.9 | -15.4 | 75 | 2473 | 72.6 | 1.8 | 0.7 |
| Europe | Netherlands | 5.4 | 52.1 | 34 | 947 | 31.1 | 1.7 | 1.2 |
| Americas | Belize | -88.6 | 17.2 | 86 | 2378 | 82.4 | 1.7 | 1.9 |
| Americas | Costa Rica | -83.9 | 10.0 | 198 | 4215 | 197.1 | 1.4 | -0.5 |
| Europe | Ireland | -8.2 | 53.2 | 26 | 989 | 21.8 | 1.2 | 3.0 |
| Europe | Finland | 26.3 | 64.5 | 19 | 664 | 18.4 | 0.7 | 0.0 |
| Oceania | Tuvalu | 179.2 | -8.5 | 23 | 1470 | 20.7 | 0.6 | 1.7 |
| Europe | Norway | 8.7 | 61.2 | 25 | 863 | 26.0 | 0.2 | -1.2 |
| Asia | Uzbekistan | 63.2 | 41.8 | 35 | 635 | 37.7 | -0.3 | -2.4 |
| Europe | Belgium | 4.7 | 50.6 | 32 | 912 | 32.1 | -0.7 | 0.6 |
| Europe | Estonia | 25.8 | 58.7 | 15 | 600 | 17.1 | -0.8 | -1.3 |
| Europe | Luxembourg | 6.1 | 49.8 | 14 | 526 | 15.9 | -0.8 | -1.1 |
| Europe | Lithuania | 23.9 | 55.3 | 17 | 642 | 18.9 | -0.8 | -1.1 |
| Europe | United Kingdom | -1.6 | 53.0 | 44 | 1286 | 45.0 | -0.8 | -0.1 |
| Europe | Latvia | 25.6 | 56.9 | 16 | 673 | 17.8 | -0.9 | -0.9 |
| Europe | Poland | 19.4 | 52.1 | 33 | 958 | 36.7 | -1.2 | -2.5 |
| Oceania | Tonga | -175.2 | -21.2 | 32 | 1907 | 34.3 | -1.7 | -0.6 |
| Asia | Georgia | 43.5 | 42.2 | 39 | 744 | 38.8 | -1.7 | 1.9 |
| Oceania | Nauru | 166.9 | -0.5 | 20 | 1386 | 22.4 | -1.9 | -0.5 |
| Europe | Switzerland | 7.9 | 46.9 | 43 | 1125 | 45.1 | -2.0 | -0.1 |
| Asia | Sri Lanka | 80.7 | 7.6 | 170 | 3192 | 171.5 | -2.1 | 0.6 |
| Asia | Pakistan | 69.4 | 30.0 | 140 | 2310 | 141.2 | -2.9 | 1.7 |
| Europe | Iceland | -18.5 | 64.8 | 7 | 459 | 12.9 | -2.9 | -3.0 |
| Europe | Andorra | 1.6 | 42.5 | 16 | 350 | 16.7 | -3.2 | 2.5 |
| Asia | Lebanon | 35.9 | 33.9 | 45 | 941 | 42.6 | -3.2 | 5.6 |
| Asia | Tajikistan | 69.4 | 38.7 | 40 | 571 | 46.0 | -3.8 | -2.2 |
| Americas | St. Lucia | -61.0 | 13.9 | 35 | 1419 | 37.4 | -4.2 | 1.9 |
| Americas | Antigua and Barbuda | -61.8 | 17.1 | 30 | 1427 | 33.8 | -4.3 | 0.5 |
| Americas | St. Kitts and Nevis | -62.8 | 17.3 | 29 | 1368 | 33.0 | -4.5 | 0.5 |
| Americas | Grenada | -61.7 | 12.1 | 34 | 1438 | 38.1 | -4.7 | 0.6 |
| Americas | Uruguay | -56.0 | -32.8 | 31 | 1485 | 36.9 | -4.7 | -1.3 |
| Africa | Sudan | 30.0 | 13.8 | 196 | 2849 | 201.2 | -5.0 | -0.2 |
| Americas | Dominica | -61.4 | 15.5 | 31 | 1471 | 36.9 | -5.2 | -0.7 |
| Americas | Barbados | -59.6 | 13.2 | 33 | 1435 | 38.8 | -5.4 | -0.4 |
| Americas | St. Vincent and the Grenadines | -61.2 | 13.2 | 33 | 1452 | 40.0 | -5.6 | -1.4 |
| Americas | United States | -98.6 | 39.6 | 512 | 8441 | 518.4 | -5.7 | -0.7 |
| Asia | Mongolia | 102.9 | 46.1 | 23 | 667 | 31.7 | -5.8 | -3.0 |
| Europe | Malta | 14.4 | 35.9 | 15 | 714 | 24.2 | -6.0 | -3.2 |
| Oceania | Kiribati | 175.0 | -1.5 | 27 | 1719 | 35.7 | -6.1 | -2.5 |
| Asia | Afghanistan | 65.2 | 33.7 | 57 | 763 | 63.8 | -6.3 | -0.5 |
| Africa | Ethiopia | 39.6 | 8.6 | 158 | 1897 | 163.2 | -6.7 | 1.5 |
| Asia | Armenia | 44.6 | 40.5 | 15 | 574 | 30.6 | -7.3 | -8.3 |
| Oceania | Samoa | -172.4 | -13.7 | 37 | 1814 | 46.6 | -7.4 | -2.2 |
| Africa | Botswana | 23.8 | -22.2 | 63 | 1413 | 70.6 | -7.5 | -0.1 |
| Asia | Cyprus | 33.2 | 35.0 | 21 | 852 | 35.3 | -8.7 | -5.5 |
| Asia | Bahrain | 50.6 | 26.0 | 30 | 861 | 38.5 | -9.2 | 0.7 |
| Americas | Trinidad and Tobago | -61.3 | 10.5 | 68 | 2203 | 77.0 | -9.5 | 0.5 |
| Asia | Qatar | 51.2 | 25.3 | 30 | 892 | 40.0 | -9.9 | -0.1 |
| Asia | Israel | 34.9 | 31.0 | 78 | 2314 | 82.2 | -10.5 | 6.3 |
| Asia | Kuwait | 47.4 | 29.5 | 35 | 997 | 44.2 | -10.5 | 1.3 |
| Africa | Namibia | 17.2 | -22.1 | 101 | 2399 | 113.8 | -10.9 | -1.9 |
| Africa | Mauritius | 57.6 | -20.3 | 49 | 2184 | 60.8 | -11.3 | -0.5 |
| Africa | Lesotho | 28.2 | -29.6 | 21 | 538 | 33.8 | -11.5 | -1.3 |
| Africa | Djibouti | 42.5 | 11.9 | 58 | 2002 | 69.6 | -12.0 | 0.3 |
| Asia | Nepal | 83.9 | 28.3 | 86 | 1592 | 101.3 | -12.0 | -3.2 |
| Africa | Chad | 18.7 | 15.4 | 67 | 1035 | 83.0 | -12.8 | -3.2 |
| Africa | Tunisia | 9.6 | 35.4 | 39 | 1152 | 52.2 | -12.9 | -0.3 |
| Oceania | Solomon Islands | 160.1 | -9.6 | 108 | 3188 | 123.8 | -14.5 | -1.3 |
| Asia | United Arab Emirates | 54.2 | 23.5 | 47 | 1397 | 62.8 | -15.0 | -0.8 |
| Africa | Comoros | 43.3 | -11.8 | 43 | 1878 | 62.2 | -15.1 | -4.1 |
| Africa | Niger | 9.4 | 17.4 | 66 | 889 | 83.3 | -15.4 | -1.9 |
| Americas | Paraguay | -58.4 | -23.2 | 42 | 1665 | 61.5 | -15.9 | -3.6 |
| Asia | Maldives | 72.9 | 3.5 | 31 | 1841 | 51.2 | -16.5 | -3.7 |
| Africa | Seychelles | 55.5 | -4.6 | 54 | 2509 | 71.1 | -16.6 | -0.5 |
| Asia | Japan | 139.1 | 36.5 | 236 | 5392 | 253.6 | -17.1 | -0.5 |
| Asia | Oman | 57.4 | 21.7 | 82 | 2143 | 98.8 | -17.6 | 0.8 |
| Africa | Libya | 18.0 | 27.0 | 38 | 902 | 58.3 | -18.3 | -2.1 |
| Americas | Chile | -69.4 | -23.4 | 67 | 1764 | 89.1 | -18.8 | -3.2 |
| Americas | Cuba | -77.8 | 21.3 | 69 | 2245 | 86.9 | -19.3 | 1.4 |
| Americas | Nicaragua | -85.0 | 12.8 | 148 | 3439 | 170.7 | -21.3 | -1.4 |
| Africa | Morocco | -5.8 | 32.7 | 66 | 1980 | 89.4 | -23.5 | 0.1 |
| Asia | Iraq | 43.8 | 33.0 | 45 | 1247 | 71.9 | -23.7 | -3.2 |
| Africa | Zimbabwe | 29.9 | -19.0 | 77 | 1846 | 110.4 | -23.9 | -9.5 |
| Americas | Jamaica | -77.3 | 18.2 | 39 | 1613 | 67.5 | -23.9 | -4.6 |
| Americas | Dominican Republic | -70.7 | 19.0 | 48 | 1822 | 75.8 | -25.2 | -2.6 |
| Oceania | Papua New Guinea | 143.5 | -5.9 | 388 | 5950 | 414.3 | -26.0 | -0.3 |
| Americas | Haiti | -72.3 | 19.1 | 55 | 1769 | 81.9 | -26.2 | -0.6 |
| Americas | Guyana | -59.0 | 4.8 | 109 | 3227 | 136.9 | -27.3 | -0.6 |
| Americas | Ecuador | -78.5 | -1.4 | 298 | 5459 | 328.6 | -27.4 | -3.2 |
| Asia | Jordan | 36.3 | 30.7 | 52 | 1806 | 87.3 | -28.0 | -7.3 |
| Africa | Algeria | 2.6 | 28.2 | 57 | 1311 | 89.3 | -29.7 | -2.5 |
| Oceania | Australia | 136.2 | -25.0 | 388 | 8758 | 420.3 | -31.3 | -1.0 |
| Africa | Kenya | 37.9 | 0.5 | 310 | 4663 | 344.7 | -31.5 | -3.2 |
| Americas | Suriname | -55.9 | 4.1 | 88 | 2876 | 124.1 | -33.0 | -3.2 |
| Americas | Honduras | -86.9 | 14.8 | 148 | 3634 | 186.4 | -33.2 | -5.2 |
| Europe | France | 2.5 | 46.6 | 75 | 2909 | 113.9 | -33.5 | -5.4 |
| Europe | Portugal | -8.1 | 40.3 | 51 | 2622 | 95.1 | -39.8 | -4.2 |
| Europe | Spain | -3.6 | 40.2 | 82 | 3543 | 131.7 | -48.1 | -1.6 |
| Asia | Saudi Arabia | 44.6 | 24.0 | 78 | 2341 | 134.4 | -52.8 | -3.6 |
| Africa | Madagascar | 46.7 | -19.4 | 218 | 4370 | 275.9 | -57.2 | -0.7 |
| Africa | Somalia | 48.3 | 9.8 | 135 | 3202 | 203.0 | -65.3 | -2.7 |
| Asia | India | 78.5 | 21.0 | 633 | 7263 | 735.5 | -100.8 | -1.7 |
| Africa | South Africa | 23.1 | -30.6 | 177 | 5715 | 303.5 | -122.4 | -4.1 |
| Asia | Philippines | 122.5 | 11.1 | 324 | 6206 | 476.0 | -144.7 | -7.3 |

**Supplementary Table S2. Top ten countries housing Data Deficient species a) overall and b) by area based on all taxa assessed in the IUCN Red List.**

| a) | | b) | | |
| --- | --- | --- | --- | --- |
| **Country** | **Data deficient species** | **Country** | **Data deficient species by land area (km^2^)** |  |
| Indonesia | 1225 | Nauru | 1.00 |  |
| Vietnam | 764 | Tuvalu | 0.77 |  |
| China | 753 | Singapore | 0.25 |  |
| Brazil | 721 | San Marino | 0.22 |  |
| Malaysia | 692 | Marshall Islands | 0.18 |  |
| India | 635 | Seychelles | 0.12 |  |
| Colombia | 611 | St. Kitts and Nevis | 0.11 |  |
| Thailand | 609 | Maldives | 0.10 |  |
| Myanmar | 607 | Grenada | 0.10 |  |
| Mexico | 552 | St. Vincent and the Grenadines | 0.08 |  |
